# Epicardial high-resolution mapping of advanced interatrial block: Relating ECG, conduction abnormalities and excitation patterns

**DOI:** 10.3389/fcvm.2022.1031365

**Published:** 2023-01-12

**Authors:** Nawin L. Ramdat Misier, Mathijs S. van Schie, Chunsheng Li, Frans B. S. Oei, Frank R. N. van Schaagen, Paul Knops, Yannick J. H. J. Taverne, Natasja M. S. de Groot

**Affiliations:** ^1^Department of Cardiology, Erasmus Medical Center, Rotterdam, Netherlands; ^2^Department of Cardiothoracic Surgery, Erasmus Medical Center, Rotterdam, Netherlands

**Keywords:** epicardial mapping, Bachmann’s Bundle, atrial fibrillation, interatrial block (IAB), ECG

## Abstract

**Background:**

Impairment of conduction across Bachmann’s Bundle (BB) may cause advanced interatrial block (a-IAB), which in turn is associated with development of atrial fibrillation. However, the exact relation between a complete transverse line of conduction block (CB) across BB and the presence of a-IAB has not been studied.

**Objective:**

The aims of this study are to determine whether (1) a complete transversal line of CB across BB established by high resolution mapping correlates with a-IAB on the surface ECG, (2) conduction abnormalities at the right and left atria correlate with a-IAB, and (3) excitation patterns are associated with ECG characteristics of a-IAB.

**Methods:**

We included 40 patients in whom epicardial mapping revealed a complete transverse line of CB across BB. Pre-operative ECGs and post-operative telemetry were assessed for the presence of (a) typical a-IAB and *de novo* early post-operative AF (EPOAF), respectively. Total atrial excitation time (TAET) and RA-LA delay were calculated. Entry site and trajectory of the main sinus rhythm wavefront at the pulmonary vein area (PVA) were assessed.

**Results:**

Thirteen patients were classified as a-IAB (32.5%). In the entire atria and BB there were no differences in conduction disorders, though, patients with a-IAB had an increased TAET and longer RA-LA delay compared to patients without a-IAB (90.0 ± 21.9 ms vs. 74.9 ± 13.0 ms, *p* = 0.017; 160.0 ± 27.0 ms vs. 136.0 ± 24.1 ms, *p* = 0.012, respectively). Patients with typical a-IAB solely had caudocranial activation of the PVA, without additional cranial entry sites. Prevalence of *de novo* EPOAF was 69.2% and was similar between patients with and without a-IAB.

**Conclusion:**

A transverse line of CB across BB partly explains the ECG characteristics of a-IAB. We found atrial excitation patterns underlying the ECG characteristics of both atypical and typical a-IAB. Regardless of the presence of a-IAB, the clinical impact of a complete transverse line of CB across BB was reflected by a high incidence of *de novo* EPOAF.

## Introduction

Bachmann’s Bundle (BB) is the main preferential route for interatrial conduction (1, 2). Data from recent studies suggest that conduction disorders at BB are involved in the pathophysiology of atrial fibrillation (AF) (3, 4), but the mechanistic relationship is yet to be elucidated. Impairment of conduction across BB causes advanced interatrial block (a-IAB), which is in turn strongly associated with development of supraventricular tachycardia, including AF (5–7).

The relationship between BB and a-IAB was substantiated by the observation that a surgical lesion across BB resulted in electrocardiogram (ECG) characteristics similar to a-IAB; namely, biphasic P waves appearing particularly in the inferior leads of the surface ECG (8). These ECG characteristics have been attributed to a caudocranial activation of the left atrium (LA) due to other dormant interatrial connection, but exact activation patterns underlying a-IAB are scarcely reported (7, 9). Introduction of typical and atypical a-IAB—subvariants of a-IAB based on alternative ECG patterns–has further complicated understanding of the excitation patterns (10).

Importantly, the relation between a complete transverse line of conduction block (CB) across BB in patients with ECG features of a-IAB has never been investigated, even though this is thought to underlie a-IAB (8). Recent reports even suggest that the site of CB is not between right atrium (RA) and LA, but rather the roof of the LA (11, 12). Epicardial mapping during cardiac surgery provides a unique methodology to examine impairment of conduction across BB on a high-resolution scale. The aims of this study are therefore to determine whether (1) a complete transversal line of CB across BB established by high resolution mapping correlates with a-IAB on the surface ECG, (2) conduction abnormalities at the right and left atria correlate with a-IAB, and (3) excitation patterns are associated with ECG characteristics of a-IAB.

## Materials and methods

### Study population

Patients (≥18 years) undergoing elective cardiac surgery with or without a history of AF were included in this current study. Exclusion criteria were hemodynamic instability, presence of an implanted pacemaker with atrial pacing, previous cardiac surgery, end-stage renal failure, or severely impaired left ventricular function. This study is part of the QUASAR (MEC 2010-054) and Halt and Reverse (MEC 2014-393) studies, which follow the declaration of Helsinki principles (13, 14). Patient characteristics were obtained from electronic medical files. Written informed consent was obtained from all patients.

### Identification of inter-atrial conduction block

**FIGURE 1:**
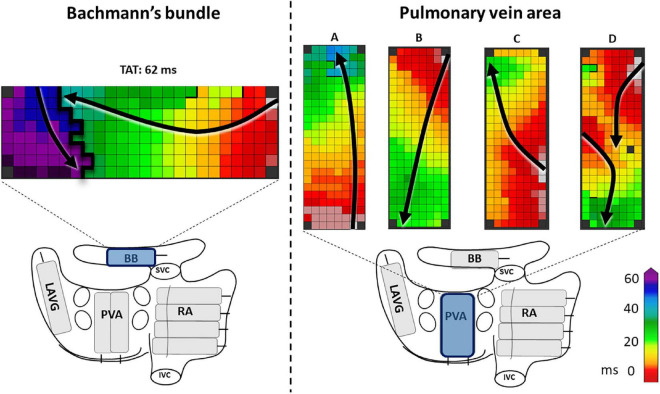
The **left panel** shows an example of a complete line of conduction block across BB, ranging from the superior border up to the inferior border. The **right panel** shows examples of activation patterns at the pulmonary vein area (PVA). In example A and C the PVA is activated in a caudocranial direction, in B and D craniocaudal direction. In examples A, B, and C the entry sites of the wavefronts are, respectively caudal, cranial and mid right-sided. In example D, there are two entry sites of the wavefront, one left-sided and one cranial. Black lines indicate conduction block. Black arrows show the direction of the wavefront. BB, Bachmann’s bundle; LAVG, left atrioventricular groove; PVA, pulmonary vein area; RA, right atrium; SVC, superior caval vein; IVC, inferior caval vein.

For this study, we solely selected patients in whom epicardial mapping revealed a complete transverse line of CB (impairing longitudinal conduction) across BB, which was defined as a continuous line of CB from the superior to the inferior border of BB (Figure 1 left panel). Subsequently, pre-operative ECGs of these patients were evaluated by two independent investigators for signs of advanced (atypical) a-IAB as described by Bayes de Luna et al. including a P wave duration ≥120 ms and biphasic P wave morphology in leads II, III and aVF (10). P wave duration was measured both manually and automatically by using custom-made MEANS-algorithm (15). We classified our selected patients into patients with a-IAB (typical or atypical a-IAB) and patients without a-IAB. Subsequently, we subdivided patients with a-IAB into patients with typical and atypical a-IAB based on the presence or absence of a biphasic P wave in lead II, respectively (10).

### Mapping procedure

Prior to initiation of extracorporeal circulation, epicardial mapping was performed during cardiac surgery at the RA, BB, and LA, consisting of the left atrioventricular groove (LAVG) and pulmonary vein area (PVA) (13). A schematic overview of the atrial mapping sites is provided in the Figure 1 lower part. Mapping was performed during sinus rhythm (SR), with either a custom-made 128- or a 192-unipolar electrode array (inter-electrode distances of both arrays: 2 mm). At every mapping site, signals were recorded during SR for 5 s, including a surface ECG, bipolar reference electrode and a calibration signal of 2 mV and 1,000 ms. Recordings were amplified with gain 1,000, sampled with a rate of 1 kHz, filtered with bandwidth 0.5–400 Hz, analog-to-digital converted (16 bits) and stored on hard disk.

### Data processing

Mapping data was analyzed by using custom-made software previously described in detail (13). Color-coded local activation time maps were constructed by annotating the steepest negative atrial deflection. Mapping locations were excluded when deflections were marked in less than 40% of the mapping array due to a poor signal-to-noise ratio. Atrial extra systolic beats were excluded. For every mapping location [RA, BB, LA (= LAVG + PVA)] total activation times (TAT) were calculated by finding the first and last activation of the corresponding mapping sites compared to the bipolar reference electrode. Similarly, total atrial excitation time (TAET) for both atria, including BB, was determined. Furthermore, the time difference between first activation at the RA and onset of LA activation (RA-LA delay) was determined.

Conduction times (CTs) were calculated by calculating the difference in local activation time between adjacent electrodes. Similar to previous studies, CB was determined as CT differences of ≥12 ms between two electrodes (16). If an area of conduction delay (CD, CTs 7–11 ms) was connected to a CB area it was labeled as a continuous CDCB area (cCDCB). The prevalence of CB was determined as the percentage CTs ≥12 ms in comparison to all CTs.

In order to elucidate the ECG characteristics of a-IAB, we investigated the activation patterns at the LA. Both entry site and trajectory of the main SR wavefronts at the pulmonary vein area (PVA) were determined, as illustrated in the Figure 1 right panel. The direction of the wavefront was assessed for caudocranial excitation (Figure 1 panel B and D) and the presence additional cranial entry sites (Figure 1 panel D).

### Detection of post-operative atrial fibrillation

Cardiac rhythms of all patients were continuously recorded from the moment of arrival on the surgical ward to the end of the fifth post-operative day using bedside monitors (Draeger Infinity™, Lübeck, Germany). Automatic algorithms were used to detect early post-operative AF (EPOAF) episodes lasting >30 s. All episodes detected by the software were cross-checked by two blinded operators in order to eliminate potential false positive registrations induced by artifacts.

### Statistical analysis

Normally distributed continuous variables were expressed as mean ± standard deviation, skewed variables as median values with interquartile ranges and categorical data as numbers and percentages. Continuous data was analyzed using the Mann–Whitney U-test or independent *t*-test and categorical data with χ^2^ or Fisher exact test when appropriate. A P-value <0.05 was considered statistically significant. All statistical analyses were performed with IBM SPSS statistics for Windows, version 25 (IBM Corp., Armonk, NY, USA).

## Results

### Baseline characteristics

A total of 40 patients were included [age: 71.5 ± 7.3 years, 30 male (75.0%)]. The indication for surgery for most patients was ischemic heart disease (*n* = 18, 45.0%), followed by aortic valve disease (*n* = 16, 40%), and mitral valve disease (*n* = 6, 15.0%). Thirteen patients (32.5%) had a history of AF; only one patient (2.5%) had atrial flutter.

Based on the aforementioned Bayes de Luna criteria, only 13 patients were classified as a-IAB (32.5%) [patients without a-IAB *n* = 27 (67.5%)]. The median time interval between the pre-operative ECGs and cardiac surgery was 15 days (interquartile range: 6–22 days). Baseline characteristics of both patient groups are described in Table 1. Patients with a-IAB did not differ from patients without a-IAB, except for age; patients with a-IAB were older than patients without a-IAB (74.9 ± 6.6 years vs. 69.9 ± 7.1 years, *p* = 0.038).

**TABLE 1:** Baseline characteristics.

	No a-IAB (*n* = 27)	a-IAB (*n* = 13)	*P*-value
Age	69.9 ± 7.1	74.9 ± 6.6	**0.038**
BMI	28.2 ± 4.5	27.6 ± 3.6	0.714
Gender (male)	18 (66.7%)	12 (92.3%)	0.124
History of SVT	9 (33.3%)	5 (38.5%)	1.000
**UHD**			
IHD	11 (40.7%)	7 (53.8%)	0.739
AVD (/ + IHD)	12 (44.4%)	4 (30.8%)	
MVD (/ + IHD)	4 (14.8%)	2 (15.4%)	
**AAD**			
Class I	1 (3.7%)	0	1.000
Class II	18 (66.7%)	10 (76.9%)	0.716
Class III	0	2 (15.4%)	0.100
Class IV	0	0	–
Hypertension	16 (59.3%)	9 (69.2%)	0.730
Dyslipedemia	15 (55.6%)	4 (30.8%)	0.141
Diabetes mellitus	6 (22.2%)	5 (38.5%)	0.451
Myocardial infarction	8 (29.6%)	2 (15.4%)	0.451
Thyroid disease	4 (14.8%)	0	0.284
LAVI	30.1 (4–39)	47 (26.3–84.3)	0.343
**Systolic LVF**			
Normal	21 (77.8%)	10 (76.9%)	1.000
Mild dysfunction	5 (18.5%)	3 (23.1%)	
Moderate dysfunction	1 (3.7%)	0	
P wave duration ≥120 ms	21 (77.7%)	13 (100%)

AVD, aortic valve disease; BMI, body mass index; IHD, ischemic heart disease; LAVI, left atrial volume index; LVF, left ventricular function; MVD, mitral valve disease; SVT, supraventricular tachycardia; UHD, underlying heart disease. AAD, antiarrhythmic drugs. A *p*-value < 0.05 was considered statistically significant.

### Relation between a-IAB and conduction properties

**FIGURE 2:**
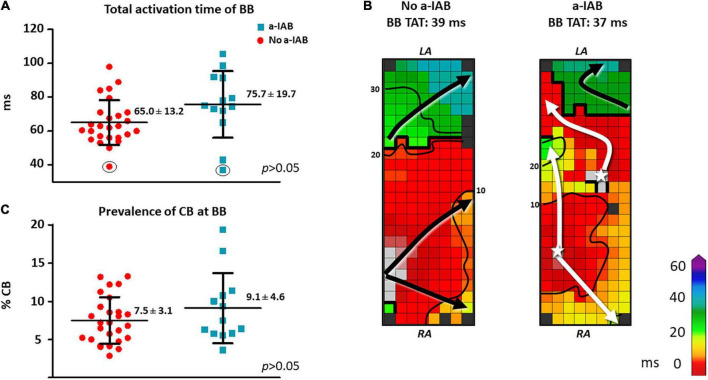
In the left panel, boxplots depict the differences in activation time **(A)** and prevalence of CB **(C)** at BB between the no a-IAB and a-IAB group. In the right panel, color-coded activation maps of BB show examples of a short TAT of BB in a patient from the no a-IAB group **(B)** and one from the a-IAB group **(B)**. See the text for further description. Activation maps are depicted with 10 ms isochrones and arrows showing the main direction of wavefront propagation. Black lines indicate an area of CB and a star represents an epicardial breakthrough wave. AT, activation time; BB, Bachmann’s bundle; CB, conduction block; IHD, ischemic heart disease; LA, left atrium; MVD, mitral valve disease; RA, right atrium.

As demonstrated by the boxplots in Figure 2A, TAT-BB did not differ significantly between patients with a-IAB and patients without a-IAB (75.7 ± 19.7 ms vs. 65.0 ± 13.2 ms, *p* = 0.051, respectively). While long TAT-BB were expected in all patients, three patients (1 a-IAB, 2 no a-IAB) had a considerably shorter TAT-BB compared to other patients. Figure 2B demonstrates color-coded activation time maps from two of these patient with short TAT-BB. The left and right color-coded maps correspond to a patients with a-IAB and a patient without a-IAB, respectively. Although both patients have a similar TAT-BB, the entry site of the SR wavefront into BB is different between the patients. In the patient without a-IAB, the earliest activation at BB is a wavefront entering in the corner of the right atrial site. In contrast, in the patient with a-IAB the earliest wavefronts emerge from the middle of BB, indicated with a white star in Figure 2. Hence, these wavefronts did not originate from the RA site of BB, indicating an obstruction between the superior RA and the RA insert site of BB, inhibiting a right-to-left activation of BB. Thus, even though the TAT-BB is short in this patient, the BB activation pattern of the patient with a-IAB still suggests an abnormal route of wavefront propagation during SR.

Besides the complete transverse line of CB across BB, multiple additional lines of CB were also present at BB. However, the amount and maximum length of CB at BB, including the complete transverse line, did not differ significantly between patients with or without a-IAB [amount of CB: 9.1 ± 4.6% vs. 7.5 ± 3.1%, *p* = 0.194; maximum CB length: 32 (24–48) mm vs. 34 (24–40) mm, *p* = 0.776, respectively], as shown in Figure 2C.

**FIGURE 3:**
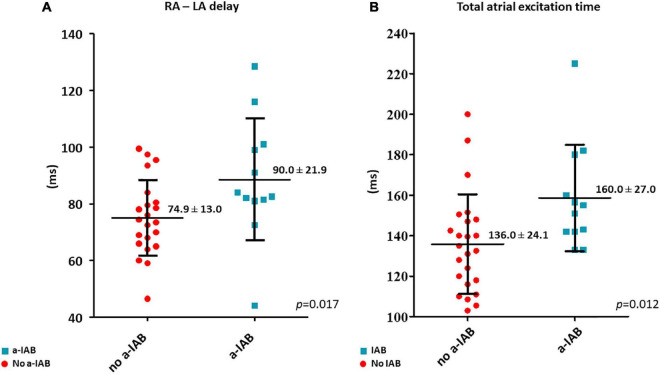
The left and right boxplots show **(A,B)** the time differences in LA activation delay and total atrial excitation time between the no a-IAB and a-IAB groups. RA, right atrium; LA, left atrium.

RA—LA delay was longer in the a-IAB group (Figure 3A; a-IAB: 90.0 ± 21.9 ms vs. no a-IAB: 74.9 ± 13.0 ms, *p* = 0.017). In addition, TAET was also longer in the a-IAB group (Figure 3B, a-IAB: 160.0 ± 27.0 ms vs. no a-IAB: 136.0 ± 24.1 ms, *p* = 0.012). However, no differences were found in TAT-RA or TAT-LA between both groups [RA: a-IAB: 82 (63.8–115.3) ms vs. no a-IAB: 82.3 (71.9–95.3) ms, *p* = 0.794; LA: a-IAB: 69.5 (60.8–90.9) ms vs. no a-IAB: 56.0 (51.0–71.0) ms, *p* = 0.096]. Thus, although there is a longer RA-LA delay and TAET in patients with a-IAB, there is no preferential site of conduction slowing in RA or LA.

Caudocranial activation of the main SR wavefront at the PVA was present in patients with a-IAB 91.7%, while only 70.8% of the patients without a-IAB had a caudocranial activation (*p* = 0.224). Caudocranial activation is thus frequently observed in patients with a-IAB, however it is not specific for a-IAB as it is also common in patients without a-IAB. Additional excitation of the PVA *via* a cranial entry was equally often present in patients with a-IAB and patients without a-IAB (41.7% vs. 58.3%, *p* = 0.345, respectively).

### Development of EPOAF

The prevalence of *de novo* EPOAF was as high as 69.2% (18/26 patients). However, the prevalence of *de novo* EPOAF did not differ between patients with and without a-IAB (62.5% vs. 72.2%, *p* = 0.667, respectively).

### Typical a-IAB vs. atypical a-IAB

In the entire study population, typical and atypical a-IAB were present in respectively, five and eight patients. Seven patients had ECGs corresponding to type 1 atypical a-IAB (87.5%) and 1 (12.5%) had type 2 atypical a-IAB. Patients with typical a-IAB pattern were older than patients with atypical a-IAB (80.1 ± 2.9 years vs. 71.7 ± 6.2 years, *p* = 0.017, respectively). Development of *de novo* EPOAF did not differ between these groups (typical a-IAB: 66.7% vs. atypical a-IAB: 60.0%, *p* = 1.000).

Patients with typical and atypical a-IAB differed in entry site of SR wavefronts at the PVA. An additional cranial entry site was never present in patients with typical a-IAB, while 71.4% patients with atypical a-IAB had a cranial entry site (*p* = 0.028). All patients with an additional cranial entry site also had caudal or mid-right entry site of the main SR wavefront. Therefore, both groups, except for one patient in the atypical a-IAB group, showed caudocranial activation of the PVA during SR.

In addition, patients with typical a-IAB had longer TAT-LA compared to patients with atypical a-IAB (84.6 ± 14.4 ms vs. 58.8 ± 16.8 ms, *p* = 0.031, respectively). As shown in Supplementary Table 1, with exception of TAT-LA, patients with typical a-IAB demonstrated similar conduction properties as patients with atypical a-IAB.

## Discussion

### Key findings

This high-resolution epicardial mapping study revealed that a-IAB is only present in a part of the patients with a complete transverse line of CB across BB. In the entire RA, LA and BB there were no differences in conduction disorders. Nevertheless, patients with a-IAB had a delayed onset of LA activation and increased TAET in comparison to patients without a-IAB. Patients with typical a-IAB showed only a single caudocranial propagating wavefront activating the PVA, and TAT-LA was increased compared to patients with atypical a-IAB. Independently of the presence of a-IAB, patients with a complete transverse line of CB at BB demonstrated a high incidence of *de novo* EPOAF.

### Pathophysiology of a-IAB

Although impaired conduction at BB may underlie a-IAB (8), only one-third of our patients had ECG characteristics of (a) typical a-IAB. We therefore provide evidence that a-IAB is not solely related to interatrial conduction at BB. The low prevalence of a-IAB in this study can be explained by the presence of alternative (preferential) interatrial conduction routes. In humans, there is significant inter-individual variation in interatrial conduction through various routes, which include conduction (1) anteriorly *via* BB, (2) posteriorly *via* myocardial pathways or bridges at the level of the right pulmonary veins (also known as fossa ovalis connections), and (3) inferiorly *via* myocardial sleeves extending from the coronary sinus to the inferior part of the LA (17–20). The SR wavefront propagates predominantly across the anterior pathway (21). In up to one-third of the patients, initial LA breakthrough during SR is observed in the fossa ovalis region, which corresponds to conduction *via* the posterior interatrial connection (21). Variability in number, location, and thickness of these interatrial connections may partly explain why some individuals develop a-IAB whereas others do not. If interatrial conduction across BB is impaired, some patients will have the posterior route as an alternative option and will avoid a-IAB on ECG. However, other patients, who lack interatrial impulse propagation in the anterior and posterior parts of the interatrial septum due to advanced remodeling, will be dependent on the inferior route for interatrial conduction. They will therefore have caudocranial activation of the LA and a-IAB on ECG.

We observed no clear preferential sites for conduction slowing in the RA and LA, though patients with a-IAB did have delayed onset of LA activation and longer TAET, which suggests that conduction is likely also impaired outside the regions that are mapped, such as other interatrial conduction pathways. Compared with healthy controls, patients with AF indeed have more often impaired conduction across the anterior and posterior interatrial connections (4, 22).

Several studies have investigated the direct relation between injury of BB and ECG changes corresponding to a-IAB (8, 23, 24). In an animal model, Waldo et al. showed that a surgical lesion across the middle of BB resulted in ECG characteristics similar to a-IAB (8). Mikhaylov et al. demonstrated that ablation at the anteroseptal RA—close to the insertion of BB–will result in a-IAB on ECG (23). Moser et al. highlighted the importance of various inputs (muscle strands) of BB onto the LA for development of a-IAB (24). BB has both superficial epicardial fibers connecting to the left atrial appendage, as well as fibers from deeper layers coursing close the right pulmonary vein. Distal injury of either tract will therefore not directly result in a-IAB. In addition, BB is also connected with other parts of the atria through various (epicardial) bundles (19). For example, a posterosuperior bundle joins the posterior part of BB from the posterior right atrial wall. A wavefront entering BB in the center of the mapping array, as observed in the example in Figure 2B, can also be explained by the finding that BB is connected to the surrounding myocardial tissue as it transverses the interatrial groove. This mid-entry wavefront presumably originates from other interatrial septal pathways and continues at BB.

### Caudocranial activation

All patients with a-IAB in our study had caudocranial activation of the PVA, except for one patient with atypical a-IAB. Moreover, patients with typical a-IAB never had an additional cranial entry site at the PVA. The electrophysiological mechanism underlying the ECG pattern seen in typical a-IAB had only been investigated in a few patients (9, 11, 21). Cosio et al. also found caudocranial activation of the LA in three patients with a-IAB during catheterization (11). In a more recent mapping study, it was demonstrated in two patients with a-IAB that the LA is activated only *via* the coronary sinus and not *via* BB or the interatrial septum (21). We now provide evidence that right to left propagation over BB is completely interrupted in patients with a complete transverse line of CB at BB and typical a-IAB, as these patients did not have a cranial entry site at the PVA region.

In 2018 Bayes de Luna et al. introduced atypical a-IAB, with a variety of p-wave morphologies in the inferior leads, but with a terminal negative component in lead aVF, which still indicates caudocranial activation of the LA (10). Bayes de Luna et al. defined three subtypes of atypical a-IAB, which include (1) the terminal component of the P wave in lead II to be “isodiphasic” (flat rather than negative), (2) the terminal component of the P wave in lead II to be “biphasic” (“negative-positive”) and (3) first component of the P-wave to be isodiphasic in leads III and aVF, but biphasic in lead II (which requires ectopic atrial rhythms as differential diagnosis). In our mapping study, we only found ECGs corresponding to type 1 (*n* = 7) and type 2 atypical a-IAB (*n* = 1). In these patients the LA was predominantly activated by a caudocranial wavefront, though a (small) wavefront entering the cranial LA was still present. The presence of an extra cranial entry site in patients with atypical a-IAB could be responsible for the different terminal component of the p-wave in II, which corroborates why these patients have atypical a-IAB on ECG instead of typical a-IAB.

### Early post-operative atrial fibrillation

While a-IAB is strongly associated with development of AF, patients with and without a-IAB did not differ in the incidence of *de novo* EPOAF (5–7). For that reason, in patients with a complete line of CB on BB, a-IAB does not seem to further contribute to development of EPOAF. Instead the presence of a-IAB in our patients may rather be an indicator of extensive (electrical and/or structural) remodeling than a culprit itself in the development of AF. However, the incidence of *de novo* EPOAF is still considerably high in these patients compared to other populations undergoing cardiac surgery (25). Patients with mitral valve disease are considered to have the highest risk of developing EPOAF with a prevalence between 29.9 and 44.1% (25). In patients with an complete line of CB on BB, almost 70% of the patients developed EPOAF, which is considerably higher than patients with mitral valve disease. Conde et al. found a similar prevalence of EPOAF (66.7%) in 36 patients with a-IAB undergoing coronary artery bypass grafting (26). In line with our findings, Teuwen et al. already demonstrated that patients with long lines of transverse CB (>12 mm) across BB had a three times higher risk to develop EPOAF (4).

Currently, various mechanisms and risk factor factors for EPOAF have been suggested, and many preventive treatments for this arrhythmia have been proposed, but the prevalence of POAF remains substantial and troublesome (27). Our results provide further insights into the development of EPOAF by characterizing its pre-existing arrhythmogenic substrate. A deeper understanding of the underlying mechanisms of EPOAF will help better identify patients at risk for EPOAF, which could stimulate novel (preventive) treatments.

### Limitations

The surface ECG was obtained prior to the mapping procedure, so there was no simultaneous registration of the surface ECG and electrograms. We did not perform endocardial mapping of the interatrial septum, as this is not possible in the current setting.

## Conclusion

In the present study, we demonstrated that a complete transverse line of CB across BB partly explains the ECG characteristics of a-IAB. Patients with a-IAB had a delayed onset of LA activation and increased TAET. We found atrial excitation patterns underlying the ECG characteristics of both atypical and typical a-IAB. Patients with typical a-IAB solely had caudocranial activation of the PVA, without additional cranial entry sites. Regardless of the presence of a-IAB, the clinical impact of a complete transverse CB at BB was reflected by a high incidence of *de novo* EPOAF.

## Data availability statement

The datasets presented in this article are not readily available because of EU privacy law. Requests to access the datasets should be directed to NG, n.m.s.degroot@erasmusmc.nl.

## Ethics statement

The studies involving human participants were reviewed and approved by the METC Erasmus MC (2010-054 and 2014-393). The patients/participants provided their written informed consent to participate in this study.

## Author contributions

NR contributed to data acquisition and analysis, manuscript drafting, and conceptual thinking. MS, CL, FO, FS, PK, and YT contributed to data acquisition and critically revising the manuscript. NG contributed to manuscript drafting and conceptual thinking. All authors contributed to the article and approved the submitted version.

## References

[1] Bachmann G. The inter-auricular time interval. *Am J Physiol.* (1916) 41:309–20. 10.4022/jafib.2234 32002116 PMC6990046

[2] van Campenhout M Yaksh A Kik C de Jaegere P Ho S Allessie M Bachmann’s bundle: a key player in the development of atrial fibrillation? *Circ Arrhythm Electrophysiol.* (2013) 6:1041–6. 10.1161/CIRCEP.113.000758 24129206

[3] Kumagai K Uno K Khrestian C Waldo A. Single site radiofrequency catheter ablation of atrial fibrillation: studies guided by simultaneous multisite mapping in the canine sterile pericarditis model. *J Am Coll Cardiol.* (2000) 36:917–23. 10.1016/s0735-1097(00)00803-2 10987620

[4] Teuwen C Yaksh A Lanters E Kik C van der Does L Knops P Relevance of conduction disorders in Bachmann’s bundle during sinus rhythm in humans. *Circ Arrhythm Electrophysiol.* (2016) 9:e003972. 10.1161/CIRCEP.115.003972 27153879

[5] Bayés de Luna A Cladellas M Oter R Torner P Guindo J Marti V Interatrial conduction block and retrograde activation of the left atrium and paroxysmal supraventricular tachyarrhythmia. *Eur Heart J.* (1988) 9:1112–8. 3208776 10.1093/oxfordjournals.eurheartj.a062407

[6] Escobar-Robledo L Bayes-de-Luna A Lupon J Baranchuk A Moliner P Martinez-Selles M Advanced interatrial block predicts new-onset atrial fibrillation and ischemic stroke in patients with heart failure: the “Bayes’ Syndrome-HF” study. *Int J Cardiol.* (2018) 271:174–80. 10.1016/j.ijcard.2018.05.050 29801761

[7] Bayés de Luna A Platonov P Cosio F Cygankiewicz I Pastore C Baranowski R Interatrial blocks. a separate entity from left atrial enlargement: a consensus report. *J Electrocardiol.* (2012) 45:445–51. 10.1016/j.jelectrocard.2012.06.029 22920783

[8] Waldo A Bush H Jr Gelband H Zorn G Jr Vitikainen K Hoffman B. Effects on the canine P wave of discrete lesions in the specialized atrial tracts. *Circ Res.* (1971) 29:452–67. 10.1161/01.res.29.5.452 5120612

[9] Bayés de Luna A Fort de Ribot R Trilla E Julia J Garcia J Sadurni J Electrocardiographic and vectorcardiographic study of interatrial conduction disturbances with left atrial retrograde activation. *J Electrocardiol.* (1985) 18:1–13. 10.1016/s0022-0736(85)80029-7 3156200

[10] Bayés de Luna A Escobar-Robledo L Aristizabal D Weir Restrepo D Mendieta G Masso van Roessel A A typical advanced interatrial blocks: definition and electrocardiographic recognition. *J Electrocardiol.* (2018) 51:1091–3. 10.1016/j.jelectrocard.2018.09.004 30497736

[11] Cosio F Martin-Penato A Pastor A Nunez A Montero M Cantale C Atrial activation mapping in sinus rhythm in the clinical electrophysiology laboratory: observations during Bachmann’s bundle block. *J Cardiovasc Electrophysiol.* (2004) 15:524–31. 10.1046/j.1540-8167.2004.03403.x 15149420

[12] Hinojar R Pastor A Cosio F. Bachmann block pattern resulting from inexcitable areas peripheral to the Bachmann’s bundle: controversial name or concept? *Europace.* (2013) 15:1272. 10.1093/europace/eut142 23787907

[13] van der Does L Yaksh A Kik C Knops P Lanters E Teuwen C Quest for the arrhythmogenic substrate of atrial fibrillation in patients undergoing cardiac surgery (QUASAR Study): rationale and Design. *J Cardiovasc Transl Res.* (2016) 9:194–201. 10.1007/s12265-016-9685-1 26935733 PMC4873535

[14] Lanters E van Marion D Kik C Steen H Bogers A Allessie M HALT & REVERSE: Hsf1 activators lower cardiomyocyt damage; towards a novel approach to REVERSE atrial fibrillation. *J Transl Med.* (2015) 13:347. 10.1186/s12967-015-0714-7 PMC4635598 26541406

[15] van Bemmel J Kors J van Herpen G. Methodology of the modular ECG analysis system MEANS. *Methods Inf Med.* (1990) 29:346–53. 2233382

[16] Lanters E Yaksh A Teuwen C van der Does L Kik C Knops P Spatial distribution of conduction disorders during sinus rhythm. *Int J Cardiol.* (2017) 249:220–5. 28888481 10.1016/j.ijcard.2017.08.067

[17] Platonov P Mitrofanova L Chireikin L Olsson S. Morphology of inter-atrial conduction routes in patients with atrial fibrillation. *Europace.* (2002) 4:183–92. 10.1053/eupc.2002.0221 12135252

[18] Mitrofanova L Ivanov V Platonov P. Anatomy of the inferior interatrial route in humans. *Europace.* (2005) 7(Suppl. 2):49–55. 16102503 10.1016/j.eupc.2005.03.014

[19] Knol W Teuwen C Kleinrensink G Bogers A de Groot N Taverne Y. The Bachmann bundle and interatrial conduction: comparing atrial morphology to electrical activity. *Heart Rhythm.* (2019) 16:606–14. 30366158 10.1016/j.hrthm.2018.10.021

[20] Platonov P. Interatrial conduction in the mechanisms of atrial fibrillation: from anatomy to cardiac signals and new treatment modalities. *Europace.* (2007) 9(Suppl. 6):vi10–6. 10.1093/europace/eum201 17959684

[21] Holmqvist F Husser D Tapanainen J Carlson J Jurkko R Xia Y Interatrial conduction can be accurately determined using standard 12-lead electrocardiography: validation of P-wave morphology using electroanatomic mapping in man. *Heart Rhythm.* (2008) 5:413–8. 10.1016/j.hrthm.2007.12.017 18313600

[22] Platonov P Yuan S Hertervig E Kongstad O Roijer A Vygovsky A Further evidence of localized posterior interatrial conduction delay in lone paroxysmal atrial fibrillation. *Europace.* (2001) 3:100–7. 10.1053/eupc.2001.0150 11333046

[23] Mikhaylov E Mitrofanova L Vander M Tatarskiy R Kamenev A Abramov M Biatrial tachycardia following linear anterior wall ablation for the perimitral reentry: incidence and electrophysiological evaluations. *J Cardiovasc Electrophysiol.* (2015) 26:28–35. 10.1111/jce.12543 25215599

[24] Moser F Rieger A Pönisch C Kottkamp H. A novel ablation approach in atrial fibrillation patients undergoing fibrotic-based substrate modification: Targeting the Bachmann’s bundle? *J Cardiovasc Electrophysiol.* (2018) 29:844–53. 10.1111/jce.13486 29537666

[25] D’Agostino R Jacobs J Badhwar V Fernandez F Paone G Wormuth D The society of thoracic surgeons adult cardiac surgery database: 2019 update on outcomes and quality. *Ann Thorac Surg.* (2019) 107:24–32. 10.1016/j.athoracsur.2018.10.004 30423335

[26] Conde D van Oosten E Hamilton A Petsikas D Payne D Redfearn D Prevalence of interatrial block in patients undergoing coronary bypass graft surgery. *Int J Cardiol.* (2014) 171:e98–9. 10.1016/j.ijcard.2013.12.002 24351418

[27] Dobrev D Aguilar M Heijman J Guichard J Nattel S. Postoperative atrial fibrillation: mechanisms, manifestations and management. *Nat Rev Cardiol.* (2019) 16:417–36. 30792496 10.1038/s41569-019-0166-5

