# Supplementary material for: Epicardial high-resolution mapping of advanced interatrial block: Relating ECG, conduction abnormalities and excitation patterns
Source: Front Cardiovasc Med. 2023 Jan 12;9:1031365. doi: 10.3389/fcvm.2022.1031365 (PMC9878276; doi:10.3389/fcvm.2022.1031365)
Supplement: Supplementary file 1 [file Table_1.docx]

Supplemental Material

Tables

Supplemental Table 1

|  | **Atypical a-IAB** | **Typical a-IAB** | **p-value** |
| --- | --- | --- | --- |
| TAET (ms) | 147.6 ± 10.1 | 175 ± 34.4 | 0.094 |
| TAT-RA (ms) | 83.4 ± 29.1 | 109.0 ± 52.5 | 0.277 |
| TAT-BB (ms) | 78.6 ± 20.0 | 71.2 ± 20.4 | 0.535 |
| TAT-LA (ms) | 58.8 ± 16.8 | 84.6 ± 14.4 | **0.031** |
| CB-RA (%) | 4.2 [2.7-5.0] | 2.9 [1.5-8.8] | 0.435 |
| CB-BB (%) | 10.2 ± 5.2 | 7.3 ± 3.0 | 0.285 |
| CB-LA (%) | 1.5 ± 0.7 | 1.8 ± 0.4 | 0.416 |
| Delta TAT RA-LA (ms) | 89.8 ± 8.6 | 90.4 ± 33.2 | 0.968 |

a-IAB= advanced interatrial block, BB= Bachman’s bundle, CB= conduction block LA= left atrium, RA= right atrium, TAET= total atrial excitation time, TAT= total activation time. P-value <0.05 are considered statistically significant
